# The new aims and scope of *Arthritis Research & Therapy*

**DOI:** 10.1186/s13075-018-1518-y

**Published:** 2018-02-07

**Authors:** Christopher D. Buckley, Harris Perlman

**Affiliations:** 1 Rheumatology Research Group, Institute for Translational Inflammation Research, College of Medical and Dental Sciences, University of Birmingham Research Laboratories, Queen Elizabeth Hospital Birmingham, Birmingham, UK; 2 Director of Clinical Research, Kennedy Institute of Rheumatology, University of Oxford, Roosevelt Drive, Headington, Oxford, UK; 3 Department of Medicine, Division of Rheumatology, Feinberg School of Medicine, Northwestern University, Chicago, USA

## Aims and scope of *Arthritis Research & Therapy*

**Fig. 1:**
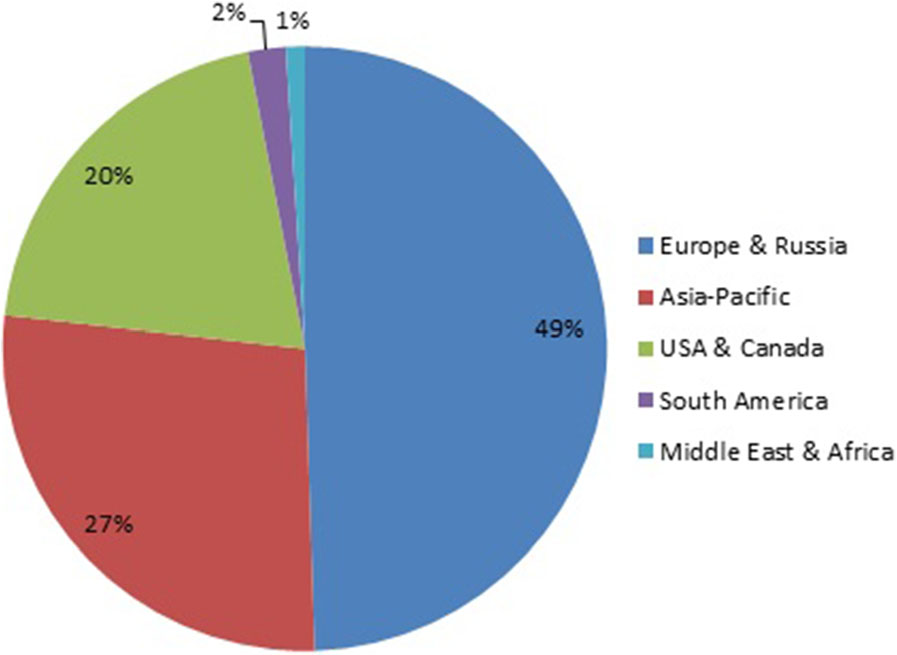
Geographical distribution of articles published in *Arthritis Research & Therapy* between September 2016 and September 2017, based on corresponding author’s country of origin
- Like all journals, we strive for fair and fast peer review. We are unusual in having two Editors-in-Chief (in two different continents) and we have established a strong group of experts who handle submissions across our six sections: Immunology and Pathology, Pharmacology and Therapeutics, Epidemiology and Clinical Trials, Bone and Cartilage Biology, Imaging and Outcomes, Genetics and Epigenetics.
- We would like to highlight emerging and exploratory studies and in particular we would like to be the journal of choice for informative science linked to clinical trials. We don’t believe that basic science can only be successfully conducted in animal models. Animals model processes; humans display the pathology.
- We would like to be a journal that publishes across the wide spectrum of autoimmune rheumatic and musculoskeletal conditions. Clinical trials are changing and new devices and diagnostic tests are emerging which will be subjected not just to the current test of “equivalence” but which will now need to be tested in randomised clinical studies. We want to be at the forefront of publishing the science that accompanies these trials, as well as the new methodologies on which they are based.

What should the aim, scope and ambition of a journal be? After two years as Editors-in-Chief of *Arthritis Research & Therapy* [1], we have been reviewing this question with our Editorial Board; not so much to produce a mission statement, but more to ensure that we continue to be relevant to our community of authors and readers. Names matter. They convey identity and uniqueness. So we spent some time discussing our journal’s name as well as its scope and what niche it should occupy in a crowded and growing field. Most members of the Board like our name. The brand is established, distinctive and we hope conveys the two themes we want to promote; research and therapy. However, we are keen to showcase not just arthritis, but the full range of autoimmune rheumatic and musculoskeletal conditions. We hope that our community will agree that we are notable for the breadth, quality and significance of emerging and exploratory studies; from pre-clinical to clinical sciences. We are particularly keen to emphasise the following points:

- We aim to be a broad, online and independent journal with a global readership and community of authors (Fig. 1) that inspires the next generation of rheumatologists. In celebration of our global reach we have commissioned commentaries from colleagues around the globe and asked them to give us their perspective of the state of musculoskeletal science in their geographic area [2]. As patients (as well as those looking after them) now actively access our journal we want to provide the highest quality of information not just to scientists, but also care workers and our patient population regardless of their location. By being open access and published under the CC BY license, all articles published in *Arthritis Research & Therapy* are freely available to communities around the world.

So in summary we hope that the following statement accurately summarizes these points and reflects the scope of our journal:

“Established in 1999, *Arthritis Research and Therapy* is an international, open access, peer-reviewed journal, publishing original articles in the area of musculoskeletal research and therapy as well as reviews, commentaries and reports.

A major focus of the journal is on the immunologic processes leading to inflammation, damage and repair as they relate to autoimmune rheumatic and musculoskeletal conditions, and which inform the translation of this knowledge into advances in clinical care.

Original basic, translational and clinical research is considered for publication along with results of early and late phase therapeutic trials, especially as they pertain to the underpinning science that informs clinical observations in interventional studies.”
